# Physical exercise interventions for perinatal depression symptoms in women: A systematic review and meta-analysis

**DOI:** 10.3389/fpsyg.2022.1022402

**Published:** 2022-12-13

**Authors:** Xueyan Liu, Guangpeng Wang, Yingjuan Cao

**Affiliations:** ^1^School of Nursing and Rehabilitation, Shandong University, Jinan, Shandong, China; ^2^Xiangya School of Nursing, Central South University, Changsha, Hunan, China; ^3^Qilu Hospital, Shandong University, Jinan, Shandong, China

**Keywords:** physical exercise, perinatal depression, systematic review, meta-analysis, randomized controlled study

## Abstract

**Background:**

The previous meta-analysis indicated that physical exercise could play a crucially therapeutic role in reducing perinatal depression symptoms in women. However, the efficacy varies across different exercise types, forms, intensities, and duration.

**Aim:**

The purpose of this study was to review and evaluate the effects of different types, forms, intensities, and duration of exercise for improving perinatal depressive symptoms.

**Design:**

A systematic review and meta-analysis.

**Methods:**

Randomized controlled trials until December 2021 were searched from seven databases, including PubMed, EMBASE, Medline, CINAHL, Web of Science, Cochrane Library, and PsycINFO. The risk of bias in eligible trials was evaluated using the Cochrane Risk of Bias tool. When high heterogeneity was tested, we used random-effects models. A funnel plot was used to assess the publication bias. This review was performed under the PRISMA guidelines, Consensus on Exercise Reporting (CERT) checklist and Cochrane Handbook. The certainty of the body of evidence was assessed using the GRADE method.

**Results:**

Of 1,573 records, 20 trials were identified in this study. The results of this review revealed that women with perinatal depression symptoms gained benefits from physical exercise [OR = 0.62, 95% CI (0.45, 0.86), *P* = 0.004; MD = −0.57, 95% CI (−0.83, −0.30), *P* < 0.0001]. Type of walking [SMD = −1.06, 95% CI (−1.92, −0.19), *P* < 0.00001], form of “Individual + group-based”exercise [SMD = −0.91, 95% CI (−0.80, −0.03), *P* = 0.04], intensity of ≥150 min per week [SMD = −0.84, 95% CI (−1.53, −0.15), *P* = 0.02], and ≥12 weeks duration [SMD = −0.53, 95% CI (−0.75, −0.31), *P* < 0.00001] seemed to generate more prominent improvement on perinatal depression symptoms.

**Conclusion:**

Physical exercise showed a significant effect on reducing perinatal depressive symptoms. This meta-analysis provides an important update on exercise’s efficacy in treating perinatal depression. Further higher quality and large-scale trials are needed to substantiate our findings.

**Systematic review registration:**

[https://www.crd.york.ac.uk/prospero/], identifier [CRD42022296230].

## Introduction

Perinatal depression, a common mental disorder in the maternal, is defined as a minor to major depressive episode during gestation or less than 12 months after delivery (Van Niel and Payne, 2020). Common symptoms include low mood, sadness, guilt or hopelessness, lack of motivation or interest, anxiety, and even suicidal ideation in a few women (Swenson et al., 2018). The pooled results in one review involved 101 studies that revealed the prevalence of perinatal depression to be 12% (Woody et al., 2017). A meta-analysis that included 95 studies in China estimated the prevalence of perinatal depression to be 16.3% and indicated that there had been an increasing trend in undeveloped areas in the past decade (Nisar et al., 2020). Perinatal depression not only impaired social and physical functioning but was a master precipitating factor in suicide (Martín-Gómez et al., 2020). Furthermore, substantial existing evidence has reported that compared with children of non-depressed mothers, children of depressed mothers were more at increased risk of emotional problems (e.g., anxiety, depression), deficit hyperactivity disorder, behavior problems, conduct disorder, poor academic performance, lower self-esteem, and social skills (Herba et al., 2016; Gentile and Fusco, 2017).

Medication is undoubtedly an essential way of clinical treatment of depression. However, the possible adverse effects of antidepressants, such as congenital disabilities, respiratory distress, and neonatal toxicity on offspring, must be considered, which causes lower treatment compliance (Latendresse et al., 2017). Treatment guidelines recommend that exercise is essential for treating perinatal depression (Cleare et al., 2015). The results from a meta-analysis have reported that significant and overall improvements in postnatal depression were recognized after immediate physical exercise intervention (Guo et al., 2020). This is probably because physical exercise can activate potential targets in the brain (Gujral et al., 2017). Currently, this intervention style is widely encouraged by virtue of its advantages of convenience, tolerance, and economy, free of stigma and side effects. However, reviewing the literature, we found that the impact of different types, forms, intensity and duration of exercise are still unclear. As an increasing number of women are expected to engage in physical activity during their perinatal period to improve mental wellbeing, clinicians, scientists, and decision-makers need to identify and implement best practices in treatment. Based on this, the primary aim of this study is to determine the effects of different exercise types, forms, intensity and duration on improving perinatal depressive symptoms to provide a basis for further development of appropriate exercise programs. The secondary aim of this study is to investigate the effects of exercise on improving perinatal depression symptoms.

## Materials and methods

This review followed PRISMA guidelines, the Consensus on Exercise Reporting (CERT) checklist and Cochrane Handbook for meta-analysis and systematic review. This study’s prospective protocol (CRD^***^) was registered on the International Prospective Register of Systematic Reviews (PROSPERO).

### Eligibility criteria

The eligibility criteria followed the population intervention control outcome study (PICOS) principles.

#### Participants

Pregnant women and postpartum (less than 12 months) mothers (≥16 years old). Perinatal depression symptoms are identified via standard measurement tools, for instance, Edinburgh Postnatal Depression Scale (EPDS) or professional psychologist.

#### Interventions

This study included trials with physical exercise as the only intervention, although the concrete mode of the intervention could vary, including yoga, walking, water sports, etc.

#### Controls

Any inactive control (e.g., standard or usual care, wait-for list) and active control (e.g., education, consulting).

#### Outcomes

Perinatal depression symptoms.

#### Design

We included randomized controlled trials (RCTs).

##### Exclusion criteria

(1) HIV, gestational diabetes, and other special population; (2) bipolar disorder, schizophrenia, current or chronic psychotic symptoms, severe PTSD, hazardous drug or alcohol use; (3) antidepressant use; (4) pregnancy after childbirth; (5) full text is not available; (6) data was insufficient to calculate the effect size; (7) reviews, conference abstracts, and study protocols.

### Search strategy

Electronic research was organized in PubMed, EMBASE, Medline, CINAHL, Web of Science, Cochrane Library, and PsycINFO from the inception to 31 December 2021. Potentially relevant papers were first identified through title and abstract searches. The detailed search strategies (Details of the search strategies were displayed in the Supplementary File 1) were as follows: “Physical exercise (Mesh)” OR “physical activity” OR “sport” OR “training” OR “endurance” OR “aerobic” OR “anaerobic” OR “resistance” OR “yoga” AND “Pregnant (Mesh)” OR “pregnancy” OR “antenatal” OR “ante-natal” OR “antepartum” OR “prenatal” OR “postnatal” OR “post-natal” OR “postpartum (Mesh)” OR “post-partum” OR “perinatal” OR “puerperium” AND “Randomized controlled trial? (Mesh)” OR “controlled clinical trial?” OR “clinical trial?” AND “Depression (Mesh)” OR “depressive” OR “mental” OR “psychological” OR “psychology” Furthermore, the reference lists of the included articles and other relevant reviews were searched manually to obtain as eligible trials as many as possible. English was the only language recognized in this study.

### Study selection

After removing duplicate articles, two review researchers (GP W, XY L) independently and sequentially screened potential studies identified as a result of the search strategy according to inclusion and exclusion criteria.

### Data extraction

Two researchers (GP W, XY L) independently extracted data for eligible literature using an agreed form designed to record data. We will contact the author for more information if the data cannot be extracted. Discrepancies between the two researchers cannot be resolved after discussion and consulting the third author. The details of the data extraction sheet included study characteristics (e.g., first author, year of publication, study design, country, sample size), population characteristics (psychological status), intervention (mode, frequency, duration, length, measure of physical activity) and outcome (perinatal depression symptoms, measure of depression).

### Study risk of bias assessment

Two researchers (GP W, XY L) independently evaluated the risk of bias for each eligible study in line with the Cochrane Handbook for Systematic Reviews of Interventions (Liao et al., 2019). The Cochrane Collaboration’s tool for assessing bias risk includes seven items, including random sequence generation (selection bias), allocation concealment (selection bias), blinding of participants and personnel (performance bias), blinding of outcome assessment (detection bias), incomplete outcome data (attrition bias), selective reporting (reporting bias) and other bias (e.g., unequal distribution among groups of potential confounders at baseline, crossovers or contamination between groups, equal, reliable, and valid outcome measurement, clear definitions of interventions). There were three options for the risk of bias under each item, including: “low risk,” “High risk,” and “Unclear risk.” Any disagreement was resolved by discussion or consulting a third researcher. In addition, we also assessed the body quality of evidence based on the GRADE system by GRADEpro^1^.

### Data synthesis

We conducted the statistical analysis using the Review Manager Software (version 5.4.1, the Cochrane Collaboration). For all dichotomous data, RRs (Mantel-Haenszel risk ratios) were calculated. The inverse variance method calculated standardized mean difference (SMD) from different measurement scales. SMD in the range of 0.2–0.5, 0.5–0.8, and >0.8 was interpreted as a small, moderate, and larger effect size (Grgic et al., 2018). And we reported 95% confidence intervals (CIs) for all outcomes. Heterogeneity between studies was assessed using the I-square (*I*^2^) value. It is generally believed that *I^2^* > 50% was considered substantial statistical heterogeneity (Liao et al., 2019), and *P* < 0.05 was considered to be statistically significant for heterogeneity (Higgins et al., 2011). If the heterogeneity was high in analyses, we used a random-effect model; otherwise, we used a fixed-effects meta-analysis. Due to the insufficient number of studies, we failed to find the source of heterogeneity by subgroup analysis. A sensitivity analysis was carried out by excluding studies one by one. When the screening results included at least three studies, publication bias was calculated and presented by funnel plot. A two-tailed *p*-value of <0.05 was considered statistically significant.

## Results

### Study selection

**FIGURE 1:**
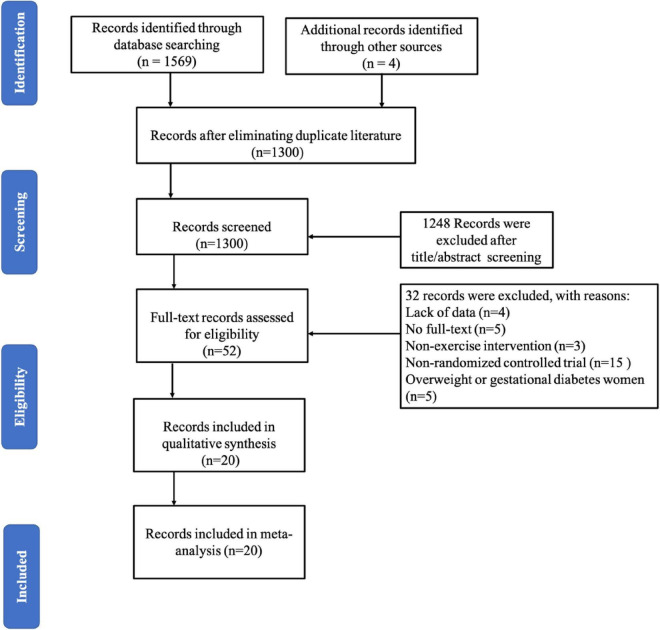
Flow diagram for the search strategy.

A PRISMA follow diagram showed the selection of papers for inclusion and exclusion (Figure 1). A total of 1,573 studies were retrieved from electronic databases, and four were added from reference lists. After excluding duplicated literature, there were 1,300 studies in the analysis, and 52 studies remained after screening the title and abstract. Finally, 20 studies (Cochrane Library, 1966; Armstrong and Edwards, 2004; Daley et al., 2008, 2015; Heh et al., 2008; Robichaud, 2009; Norman et al., 2010; Robledo-Colonia et al., 2012; Songøygard et al., 2012; Haruna et al., 2013; Buttner et al., 2015; Davis et al., 2015; El-Rafie et al., 2016; Shelton, 2016; Coll et al., 2019; Vargas-Terrones et al., 2019; Özkan et al., 2020; Navas et al., 2021; Teychenne et al., 2021) were eligible in this systematic review and performed meta-analysis after full-text review.

### Study characteristics

#### Study design and setting

Eligible study characteristics are presented in Table 1. Of these included studies reported between 2004 and 2021, only one was a pilot trial. A total of 2,866 women were in this analysis (intervention group: *n* = 1342, control group: *n* = 1,524). In this review, studies from 11 countries around the world, including the USA (*n* = 5), Australia (*n* = 3), the UK (*n* = 2), China (*n* = 2), Spain (*n* = 2), Colombia (*n* = 1), Norway (*n* = 1), Egypt (*n* = 1), Brazil (*n* = 1), Turkey (*n* = 1), and Japan (*n* = 1).

**TABLE 1:** Characteristics of randomized control trials included in the current meta-analysis.

References	Country	Study design	Target sample	Sample size (T/C)	Intervention description	Control description	Measure tools	Measurement time point	Outcome	Intervention length
Uebelacker et al. (2016)	USA	Pilot RCT	Pregnant women(12-26 weeks) with depression	Intervention: *n* = 12 Control: *n* = 8	Yoga	Mom-Baby Wellness Workshop (MBWW)	EPDS	Baseline 3 weeks (From baseline) 6 weeks (From baseline) 9 weeks (From baseline)	Prenatal depression	9 weeks
Daley et al. (2015)	UK	RCT	Postpartum women (within 6 months) with depression	Intervention: *n* = 47 Control: *n* = 47	Moderate intensity exercise + Usual care	Usual care	EPDS	Baseline 6 months (From baseline) 12 months (From baseline)	Postnatal depression	6 months
Davis et al. (2015)	USA	RCT	Pregnant women (up to 28 weeks) with depression	Intervention: *n* = 23 Control: *n* = 23	Yoga + Usual care	Usual care	EPDS	Baseline Midpoint Post-intervention	Prenatal depression	8 weeks
Robledo-Colonia et al. (2012)	Colombia	RCT	Universal pregnant women (16–20 weeks)	Intervention: *n* = 37 Control: *n* = 37	aerobic exercise + Usual care	Usual care	CES-D	Baseline 3 months (From baseline)	Prenatal depression	3 months
Norman et al. (2010)	Australia	RCT	Universal postpartum women (within 6–10 weeks)	Intervention: *n* = 37 Control: *n* = 37	Hospital based group exercise with their babies + Usual care	Written educational + usual care	EPDS	Baseline 8 weeks (From baseline) 12 weeks (From baseline)	Postnatal depression	8 weeks
Songøygard et al. (2012)	Norway	RCT	Universal Pregnant women (up to 18 weeks)	Intervention: *n* = 429 Control: *n* = 426	Exercise	Usual care	EPDS	3 months (From baseline)	Prenatal depression	12 weeks
El-Rafie et al. (2016)	Egypt	RCT	Universal pregnant women (in second trimester)	Intervention: *n* = 50 Control: *n* = 50	Exercise + Uusual care	Usual care	CES-D	Baseline 3 months (From baseline)	Prenatal depression	12 weeks
Heh et al. (2008)	China (Taiwan)	RCT	Postpartum women (4 weeks) with depression	Intervention: *n* = 40 Control: *n* = 40	Based home and hospital exercise + Usual care	Usual care	EPDS	Baseline 5 months (Postpartum)	Postnatal depression	3 months
Navas et al. (2021)	Spain	RCT	Universal pregnant women (14–20 weeks)	Intervention: *n* = 148 Control: *n* = 146	Aerobic water exercise + Usual care	Usual care	EPDS	Baseline 1 months (Postpartum)	Postnatal depression	3 times weekly, for 5 months
Coll et al. (2019)	Brazil	RCT	Universal pregnant women (16–20 weeks)	Intervention: *n* = 213 Control: *n* = 426	Exercise	Usual care	EPDS	5 weeks (From baseline) 3 months (Postpartum)	Prenatal depression Postnatal depression	16 weeks
Buttner et al. (2015)	USA	RCT	Universal postnatal women (within 12 months)	Intervention: *n* = 28 Control: *n* = 29	Yoga + Uusual care	Wait-list + usual care	HDRS	Baseline 2 weeks (From baseline) 4 weeks (From baseline) 6 weeks (From baseline) 8 weeks (From baseline)	Postnatal depression	8 weeks
Daley et al. (2008)	UK	RCT	Postnatal women (within 12 months) with depression	Intervention: *n* = 20 Control: *n* = 18	One to one exercise + Uusual care	Usual care	HDRS	Baseline 12 weeks (From baseline)	Postnatal depression	12 weeks
Teychenne et al. (2021)	Australia	RCT	Postnatal women (within 3–9 months) with depression	Intervention: *n* = 32 Control: *n* = 30	Home-based physical exercise + Usual care	Usual care	EPDS	Baseline 4 weeks (From baseline) 8 weeks (From baseline) 12 weeks (From baseline)	Postnatal depression	12 weeks
Vargas-Terrones et al. (2019)	Spain	RCT	Universal pregnant women (within 16 weeks)	Intervention: *n* = 70 Control: *n* = 54	Hospital-based exercise + Usual care	Usual care	CES-D	Baseline 38 weeks (From baseline) 6 weeks (Postpartum)	Prenatal depression; Postnatal depression	22 weeks
Özkan et al. (2020)	Turkey	RCT	Postnatal women (within 12 months) with depression	Intervention: *n* = 40 Control: *n* = 40	Exercise + Usual care	Usual care	EPDS	Baseline 4 weeks (From baseline)	Postnatal depression	4 weeks
Armstrong and Edwards (2004)	Australia	RCT	Postnatal women (within 6 weeks) with depression	Intervention: *n* = 9 Control: *n* = 10	Pram-walking exercise + Usual care	Social support	EPDS	Baseline 6 weeks (From baseline) 12 (From baseline)	Postnatal depression	12 weeks
Ko et al. (2008)	China (Taiwan)	RCT	Universal postnatal women (within 12 months)	Intervention: *n* = 31 Control: *n* = 30	Low-intensity exercise + Usual care	Usual care	CES-D	Baseline 3 weeks (From baseline)	Postnatal depression	3 weeks
Shelton (2016)	USA	RCT	Postnatal women (4–6 weeks) with depression	Intervention: *n* = 3 Control: *n* = 3	Stroller walking	Usual care	EPDS	Baseline 6 weeks (From baseline)	Postnatal depression	6 weeks
Robichaud (2009)	USA	RCT	Postnatal women (within 12 months) with depression	Intervention: *n* = 25 Control: *n* = 23	Home-based walking	Wait-list + Usual care	EPDS	Baseline 6 weeks (From baseline)	Postnatal depression	6 weeks
Haruna et al. (2013)	Japan	RCT	Universal postnatal women (within 2 months)	Intervention: *n* = 48 Control: *n* = 47	Exercise + Usual care	Usual care	EPDS	Baseline 4 months (Postpartum)	Postnatal depression	4 weeks

EPDS, edinburgh postnatal depression scale; CES-D, center for epidemiological studies depression scale; HDRS, hamilton depression rating scale.

#### Characteristics of participants

Eight studies included pregnant women, all beginning in the second trimester of pregnancy, and 12 studies included mothers during postpartum within 1 year. In addition, 10 studies included eligible participants with perinatal depression symptoms at baseline, with one study based on QIDS-SR16 (The 16-Item quick inventory of depressive symptomatology, 7–20 points), one study based on ICD (International Classification of Diseases), one study based on PHQ-9 (Patient Health Questionnaire-9, ≥9 points), two studies both based on EPDS (>12 points) and five studies in sequence based on EPDS (≥9 points, ≥10 points, >10 points, >13 points, >7 points).

#### Characteristics of interventions

Exercise types assessed in this review included comprehensive physical exercise (*n* = 11), yoga (*n* = 3), walking (*n* = 2), exercise with their babies (*n* = 1), and aerobic water exercise (*n* = 1), which professional yoga instructor or physical therapist taught. Comprehensive physical exercise interventions principally included warm-ups, aerobics, strength, etc. The form of the intervention had “individual-based,” “group-based,” and “individual + group-based.” In terms of perinatal depression measurement tools, 14 studies used EPDS (Edinburgh postnatal depression scale), four studies used CES-D (Center for Epidemiological Survey, Depression Scale), and two studies used HDRS (Hamilton depression scale). Regarding the intensity of physical exercise, participants mainly carried out three weekly sessions, with one session for 60 min. Two studies clearly defined physical exercise intensity as low to moderate. Studies primarily focused on short-term effects ranging from 3 weeks to 6 months, with 12 weeks being the most common. The participants in the control group mainly received usual care (*n* = 15) or waited for a list (*n* = 2). At the same time, one study performed “Mom-Baby Wellness Workshop,” written education, and social support. As for participants’ adherence or satisfaction to physical exercise, 10 studies did not report any information about this content.

### Risk of bias in studies

**FIGURE 2:**
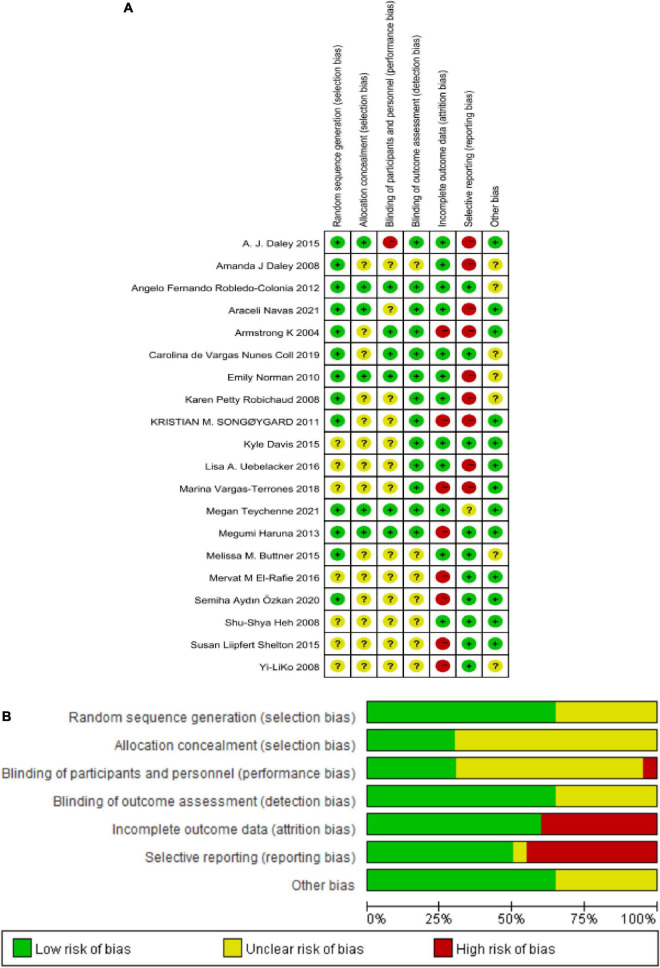
**(A)** Reviewer’ judgments about each risk of bias item for each included study. **(B)** Review authors’ judgments about each risk of bias item presented as percentages across all included studies.

The risk of bias in these enrolled studies is shown in Figures 2A,B. The methodological quality of these studies was most at low performance. Regarding these studies, 65% (13/20) studies had generated adequate random sequences conducted mainly by computer-generated number or randomization block method, 70% (14/20) studies didn’t describe the allocation concealment methods. All participants and those delivering the intervention were aware of the group allocation, and it was difficult for them to be blind. Only 30% (3/20) and 25% (5/20) of studies had a low risk of performance and detection bias. Nearly half of these studies (40%, 8/20) were at a high risk of attrition bias, in that missing outcome data was not reported. Rarely studies with available protocols, and 50% (10/20) study at low risk of reporting bias. For other biases, most studies (13/20) were at low risk. Based on the available information, the seven original studies could not judge whether they had other biases. Details of the quality ratings were displayed in the Supplementary File 1.

### Effects of physical exercise on perinatal depression symptoms

**FIGURE 3:**
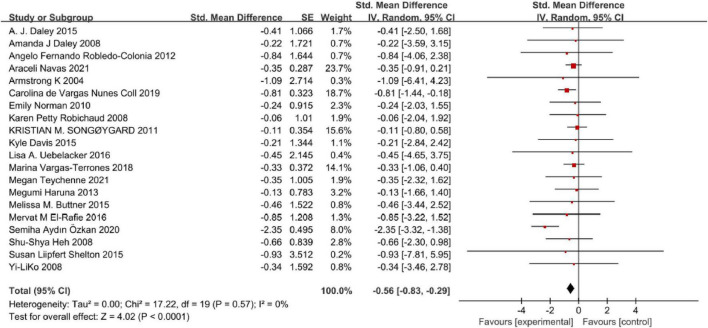
Frost plot for the effect of physical exercise intervention.

Twenty studies were available and quantitatively synthesized. Among these studies, the outcomes of 16 studies for perinatal depression symptoms were presented as continuous data. Four studies were conducted in dichotomous data (Figure 3). As dichotomous data, the pooled results showed that the risk of perinatal depression in the physical exercise intervention group was the significantly small compared control group [OR = 0.62, 95% CI (0.45, 0.86), *P* = 0.004], and without heterogeneity using fixed-effects model (Chi = 1.09, df = 3, *I*^2^ = 0, *P* = 0.78). Similarly, as continuous data, the pooled forest plot demonstrated that physical exercise can generate prominent moderate effect size on reducing perinatal depression symptoms [SMD = −0.57, 95% CI (−0.83, −0.30), *P* < 0.0001], and with high heterogeneity (Chi = 56.87, df = 15, *I*^2^ = 74%, *P* < 0.00001) using random-effects model. We further analyzed the certainty of evidence (Supplementary File 1) for all outcomes. Evidence certainty was considered low regarding comprehensive exercise, form of group and individual + group, ≥150 min per week, and exercise duration of 3–4 weeks, which was closely related to the quality of the original study. While the certainty of evidence for other outcomes was considered moderate.

### Type

**FIGURE 4:**
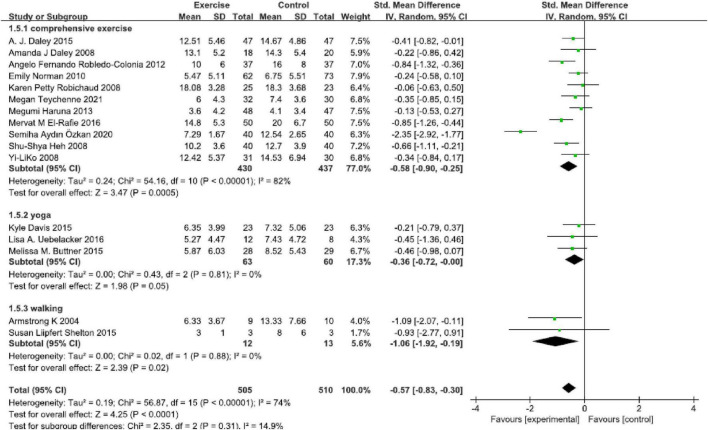
Forest plot of the perinatal depression symptoms scores at different physical exercise intervention types.

In all, 11 studies (Figure 4) reported comprehensive physical exercise [SMD = −0.58, 95% CI (−0.90, −0.25), *P* < 0.00001, *I*^2^ = 82%]. Only three studies performed yoga [SMD = −0.36, 95% CI (−0.72, 0.00), *P* = 0.005, *I*^2^ = 0], walking was presented in two studies [SMD = −1.06, 95% CI (−1.92, −0.19), *P* < 0.00001, *I*^2^ = 0], and total SMD was −0.57 [95% CI (−0.83, −0.30), *P* = 0.31, *I*^2^ = 74%]. This subgroup analysis found that various physical exercise types were superior to the control group, and there was no statistically significant subgroup difference.

### Form

**FIGURE 5:**
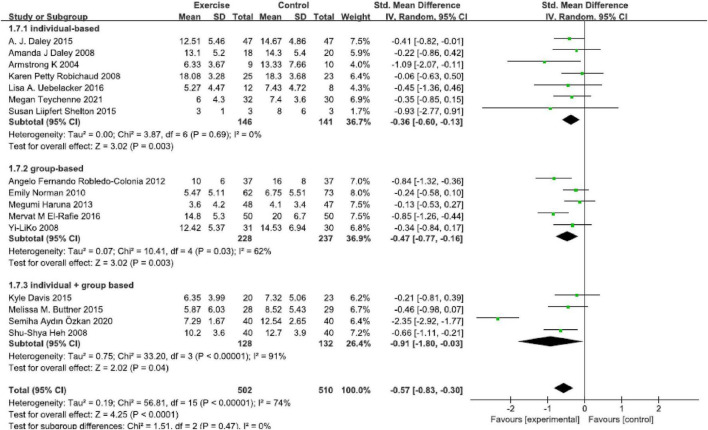
Forest plot of the perinatal depression symptoms scores at different physical exercise forms.

Sixteen studies were included in this analysis (Figure 5), and a significant effect was found in all physical exercise forms that included “individual-based [SMD = −0.36, 95% CI (−0.60, −0.13), *P* = 0.003, *I*^2^ = 0, seven studies],” “group-based [SMD = −0.47, 95% CI (−0.77, −0.16), *P* = 0.0.003, *I*^2^ = 62%, five studies]” and “individual + group based [SMD = −0.91, 95% CI (−0.80, −0.03), *P* = 0.04, *I*^2^ = 74%, four studies].” The “individual + group based” exercise found a statistically significant high effect size.

### Intensity

**FIGURE 6:**
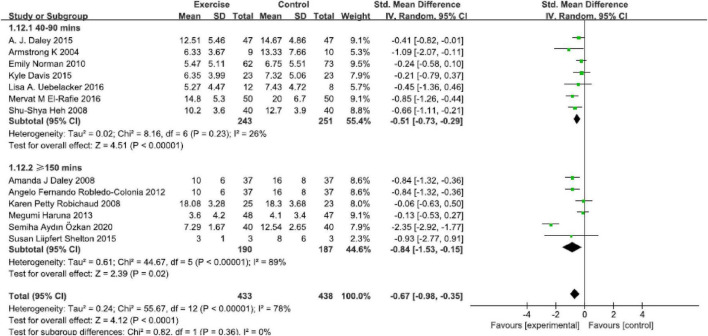
Forest plot of the perinatal depression symptoms scores at different physical exercise intervention intensities.

This analysis assessed the optimal physical exercise intensity on reducing perinatal depression symptoms (Figure 6). A significant remission was found in 40–90 min per week [SMD = −0.51, 95% CI (−0.73, −0.29), *P* < 0.00001, *I*^2^ = 26%, seven studies] and ≥150 min per week [SMD = −0.84, 95% CI (−1.53, −0.15), *P* = 0.02, *I*^2^ = 89%, six studies]. The present result in this analysis indicated that intensity of ≥150 min per week seems to be recommended.

### Duration

**FIGURE 7:**
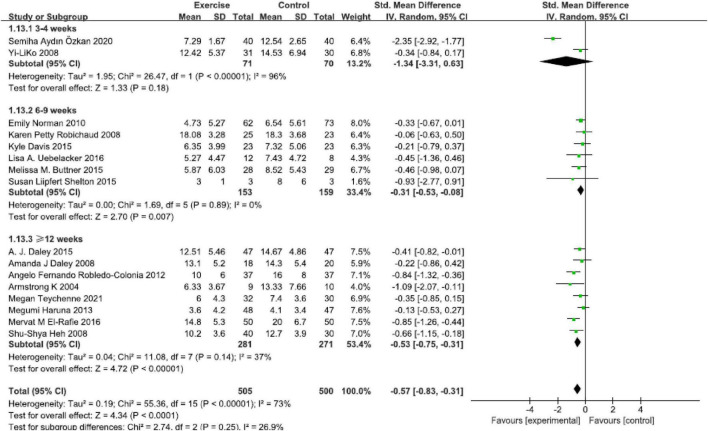
Forest plot of the perinatal depression symptoms scores at different physical exercise intervention duration.

Based on 16 studies, results revealed that intervention duration in 6–9 weeks group [SMD = −0.31, 95% CI (−0.53, −0.08), *P* = 0.007, *I*^2^ = 0, six studies], and ≥12 weeks group [SMD = −0.53, 95% CI (−0.75, −0.31), *P* < 0.00001, *I*^2^ = 37%, eight studies] all presented significant effect compared with control group. No statistically significant reductions were found in the 3–4 weeks group [SMD = −1.34, 95% CI (−3.31, −0.63), *P* = 0.18, *I*^2^ = 96%, two studies] (Figure 7). The reduction of depressive symptoms was related to the duration of exercise intervention.

### Publication bias and sensitivity analysis

**FIGURE 8:**
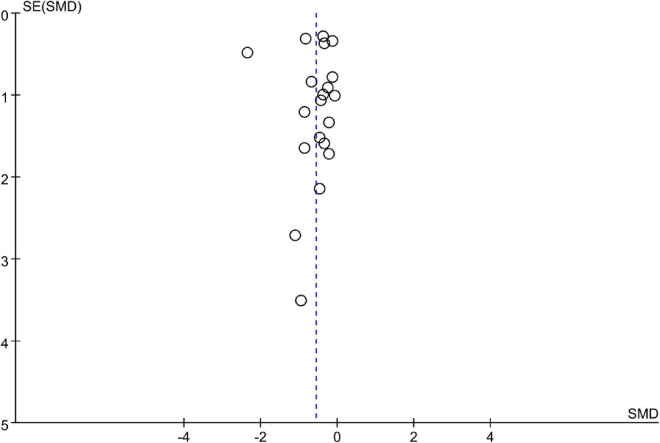
The funnel plot assesses publication bias.

The funnel plot showed no obvious asymmetry in 20 studies (Figure 8). A sensitivity analysis was performed by excluding a single study each time and found a similar finding.

## Discussion

As far as we know, there is no meta-analysis to study the effects of different exercise types, forms, intensity and duration on improving perinatal depression. In this systematic review and meta-analysis, we synthesized studies and assessed the effect size of exercise-focused interventions on perinatal depression symptoms for women. Overall, 20 randomized controlled trials were included in this meta-analysis, containing a total of 2,866 participants who were from 11 countries, and 14 trials were performed in developed countries.

The results of our study reinforced previous discoveries (Broberg et al., 2017; Coll et al., 2019) that women can benefit from physical exercise, and significant improvements in perinatal depressive symptoms were observed. Recent studies even suggested that physical exercise may become a potential alternative to antidepressants or produce synergistic effects in reducing depression symptoms (López-Torres Hidalgo, 2019; Guerrera et al., 2020). However, drug-based therapy exhibited an undeniable and substantial impact on perinatal depression. However, about 70% of women experienced side effects of antidepressants (Marasine et al., 2020) which can increase the risk of preterm birth, autism, or intellectual disability in offspring (Viktorin et al., 2018). Therefore, women felt reluctant to take antidepressants which caused low compliance (Zhao et al., 2020). Also, compared to non-pharmacology interventions such as cognitive-behaviour therapy (CBT), the physical exercise showed powerful potential strengths (Hedman-Lagerlöf et al., 2021). It is more accessible to those women with low income, learning understanding difficulties, less education, or language barriers (Van Lieshout et al., 2021). The mechanism by which exercise impacts mood is not entirely understood. Perhaps it can be attributed to the fact that physical exercise can reshape the brain structure, activate the brain targets, reduce neurotransmitter efficiency, and maintain the volume of prefrontal cortical and hippocampal (Maass et al., 2015; Thomas et al., 2016) to improve mood. Furthermore, another reason is that physical exercise can mitigate depressive mood by improving body shape (Knapen et al., 2015). Physical exercise has many benefits, and it also aids in glycemic control (Dodd et al., 2019). Although there are several advantages to performing physical exercise (Mohammadi et al., 2015; Broberg et al., 2021), it is worth noting physical exercise is not suitable for all women. Gestational hypertension, preeclampsia, incompetent cervix, placenta previa, etc., were common contraindications, and women who had performed cesarean were not advised to exercise within six weeks after delivery (Hinman et al., 2015). However, since the safety was not reported in the included studies, we may not be able to infer that exercise is safe for perinatal women. This review excluded cointerventions, and only exercise interventions were analyzed, which was more helpful in accurately evaluating the final effect of exercise, and results in this review assure high reliability.

In our meta-analysis, physical exercise mainly included comprehensive exercise, yoga, and walking, which all significantly reduced or prevented perinatal depressive symptoms. Comprehensive exercise, one of the most common types, is actually a series of aerobic exercises (Özkan et al., 2020). The comprehensive exercises in this study included different combinations of free exercise, stretching, power training, etc., effectively reducing perinatal depressive symptoms. This type of exercise may require adequate exercise space, complete exercise facilities and professional supervision. Therefore, it is not an appropriate choice for mothers who prefer to exercise at home. Yoga is also a popular type of exercise for perinatal women. Maternal yoga was different from other types of aerobic exercises, and it mainly included breathing training, body posture adjustment, meditation, muscle relaxation, etc. (Zhu et al., 2021). In a recent study, more than a 21 million Americans practiced yoga (Field, 2016), and numerous studies have identified that yoga, a popular exercise in women, may play a specific role in alleviating depressive mood and improving sleep problems and is also beneficial for stress management (Davis et al., 2015; Uebelacker et al., 2016). With the development of information technology, yoga classes for mothers can be easily accessed on mobile phones via Internet terminals, which is expected to become the mainstream type of exercise for women in the future. Walking is easier to develop and implement, especially for women in low-middle-income areas. Thus, regular walking was undoubtedly a suitable and acceptable choice to relieve mental disorders (depression, anxiety, etc.) compared to other non-pharmaceutical therapies (Saligheh et al., 2017). Moreover, walking can also enhance self-esteem, resilience, psychological wellbeing, etc. (Kelly et al., 2018; Song et al., 2018). In one experimental study, sedentary pregnant women who participated in brisk walking for at least 90 min a week had a significantly lower risk of developing prenatal depression (Taniguchi and Sato, 2016). Comprehensive exercise, yoga and walking are often used to improve the depressive symptoms of perinatal women, and there is no statistical difference in the effect value. Although walking has the best performance in improving perinatal depression, since the number of effective studies is only two, we cannot be sure that walking is better than the other two exercise types, and further investigation is needed.

In this review, “individual + group-based” exercise is more helpful in improving the depressive symptoms of perinatal women than individual-based or groused-based exercise alone. Group-based exercise can give full play to face-to-face supervision, which can enhance compliance and the effectiveness of interventions compared with unsupervised exercise (McCurdy et al., 2017). Additionally, participants who exercise in such an environment will likely receive more social support from peers or instructors (Fellmeth et al., 2017; Nisar et al., 2020). Inadequate social support was a significant risk factor for perinatal depression and a barrier to performing exercise (Morres et al., 2022). However, this form often requires sufficient exercise space. At the same time, it requires the higher organizational ability of leaders because it needs to negotiate the time and place of all participants. The form of individual-based exercise can enhance the subjective initiative of participants and avoid participation resistance caused by stigma (Zhang et al., 2019). For primary healthcare institutions with limited resources, combining group and individual exercise may be a more feasible option because more participants can be treated in a short time.

The rate of improvement of perinatal depression symptoms is closely related to the intensity of physical exercise. However, although physical exercise plays an important role in reducing perinatal depression symptoms, there is still only limited information about the optimum exercise intensity, and further work is required to explore it in detail (Dipietro et al., 2019). In this study, intensity for ≥150 min per week showed a higher effect on reducing perinatal depressive symptoms. A recent meta-analysis reported a similar finding that moderate-intensity aerobic exercise for ≥150 min per week and especially for an average of 168 min per week, was a recognized physical exercise intensity (Morres et al., 2022). This result also agreed with one previous research that recommended 30–60 min of exercise three times a week (Dipietro et al., 2019). Different intensities of exercise may influence the optimal effects (Navas et al., 2021), and another study even suggested that the higher the intensity of physical exercise, the greater relief of depressive symptoms (Davenport et al., 2018). Sustained high-intensity exercise can significantly increase VO2 max during pregnancy, and pregnant women were also encouraged to engage in moderate-intensity exercise regardless of their previous exercise habits (Dipietro et al., 2019). However, it isn’t easy to directly define exercise intensity, and all the included studies used exercise time to describe it, which may cause differences in understanding.

The duration of physical exercise varies widely, and the optimal duration also requires confirmation. Our results showed that a duration of ≥12 weeks generated a moderate effect on reducing perinatal depressive symptoms, consistent with a previous study (Carter et al., 2019). Another review suggested that a longer duration exhibited more significant results, and it took at least 4 weeks to reduce depression symptoms (McCurdy et al., 2017). Since exercise during the first trimester increases the risk of miscarriage, it is generally recommended that exercise be gradually introduced from the second trimester and help with delivery. Short exercise duration may restrict the accurate assessment, and the long-term benefits cannot be guaranteed. Further prolonged intervention duration is needed to compensate for the present study’s limitation.

## Strengths and limitations

This systematic review and meta-analysis assessed the effects of physical exercise on perinatal depression symptoms in women. This meta-analysis performed a subgroup analysis to investigate the impact of different depressed severity, exercise type, intensity, and form. And our study provides more specific references for the implementation or formulation of the exercise intervention programs. In addition, randomized controlled trials with only physical exercise interventions were included in this meta-analysis, which seems more effective than cointerventions.

Meanwhile, there are several limitations to our study. Firstly, partial subgroups with available studies for meta-analysis were relatively limited, which may affect the accuracy of the results. Secondly, without considering the baseline exercise habits and dose of exercise, the effect of exercise on reducing perinatal depression symptoms may be misestimated. Thirdly, outcome measures were inconsistent, and CED-S and HDRS were unsuitable for measuring perinatal depression. Fourth, studies had not described screening methods for exercise contraindications, and the occurrence of exercise safety incidents was also unclear. Fifth, primary studies in this review were from developed countries, and the performance of exercise intervention in developing countries cannot be effectively clarified. In addition, the majority of studies have methodological problems. Especially most studies in this review did not clearly describe the method of allocation concealment, which made it impossible to judge the randomness. Finally, we cannot guarantee the identification of all studies for various reasons. Given the above limitations, the results of this review should be interpreted with caution.

## Implications for future research and practice

Some implications in this review for future trials were as follows. Firstly, most trials have reported the effects of exercise interventions on women with perinatal depressive symptoms. However, the same exercise intervention program may have different effects on participants with different depression severity (e.g., minor, moderate, major) (Morres et al., 2022). Therefore, researchers should explore more refined and suitable exercise intervention programs according to the different severity of depression, which can prompt intervention to achieve the best performance. From a public health perspective, along with the rising global burden of disability attributed to depression, costs of depression care are rising, and adverse influences are identified, providing the impetus to push our attention from treatment to prevention (Šebela et al., 2018). Therefore, it is necessary to verify the effectiveness of physical exercise in preventing perinatal depression among the general population. Secondly, studies should clearly define the participants who are suitable for exercise in advance, which can avoid decreasing exercise safety due to ignoring the contraindications of exercise. This is essential to improve the acceptability and satisfaction of the intervention. Thirdly, exercise type, intensity, duration, and form are all crucial information to accurate the replicability and practicability of intervention. However, at present, evidence in these aspects is insufficient. Further studies should confirm the optimal exercise mode, intensity, etc., which may contribute to more standard and better exercise intervention protocols. Fourthly, the long-term effects of physical exercise intervention are worth paying more attention to. Finally, given the quality and reliability, researchers should strictly follow the reporting guidelines for randomized controlled trials in describing studies.

## Conclusion

Physical exercise is an effective and cost-effective approach to improving depressive symptoms. This meta-analysis provides an important update on exercise’s efficacy in treating perinatal depression. Evidence from 20 randomized controlled trials has demonstrated that physical exercise generated a moderate effect size on alleviating perinatal depression symptoms in women. Type of walking, the form of “Individual + group-based,” the intensity of ≥150 min per week and duration of ≥12 weeks seemed to be more suitable options. And higher-quality studies will be needed to support these findings.

## Data availability statement

The raw data supporting the conclusions of this article will be made available by the authors, without undue reservation.

## Author contributions

XL, GW, and YC: study conception and design, data collection, data analysis, interpretation, drafting of the manuscript, and critical revision of the manuscript. All authors contributed to the article and approved the submitted version.

## Footnotes

1
https://www.gradepro.org/
