# Supplementary material for: Physical exercise interventions for perinatal depression symptoms in women: A systematic review and meta-analysis
Source: Front Psychol. 2022 Dec 13;13:1022402. doi: 10.3389/fpsyg.2022.1022402 (PMC9792692; doi:10.3389/fpsyg.2022.1022402)
Supplement: Supplementary file 1 [file Data_Sheet_1.docx]

**Retrieval strategy**

*PubMed:* ("randomized controlled trials as topic"[MeSH Terms] OR ("clinical trial"[Title/Abstract] OR ("randomized controlled trial"[Title/Abstract] OR "controlled clinical trial"[Title/Abstract]))) AND ("Depression"[Title/Abstract] OR "depressive"[Title/Abstract] OR "mental"[Title/Abstract] OR "psychological"[Title/Abstract] OR "psychology"[Title/Abstract] OR ("depressive disorder"[MeSH Terms] OR "Depression"[MeSH Terms])) AND ("post-partum"[Title/Abstract] OR "perinatal"[Title/Abstract] OR "puerperium"[Title/Abstract] OR "postpartum period"[MeSH Terms] OR ("pregnancy"[Title/Abstract] OR "antenatal"[Title/Abstract] OR "ante-natal"[Title/Abstract] OR "antepartum"[Title/Abstract] OR "prenatal"[Title/Abstract] OR "pregnant"[Title/Abstract] OR "postnatal"[Title/Abstract] OR "post-natal"[Title/Abstract] OR "postpartum"[Title/Abstract]) OR "gravidity"[MeSH Terms]) AND ("physical activity"[Title/Abstract] OR "physical exercise"[Title/Abstract] OR "sport"[Title/Abstract] OR "training"[Title/Abstract] OR "endurance"[Title/Abstract] OR "aerobic"[Title/Abstract] OR "anaerobic"[Title/Abstract] OR "resistance"[Title/Abstract] OR "yoga"[Title/Abstract] OR "exercise"[MeSH Terms])

*Medline:* (AB Physical exercise OR AB physical activity OR AB sport OR AB training OR AB endurance OR AB aerobic OR AB anaerobic OR AB resistance OR AB yoga) AND (AB Pregnant OR AB pregnancy OR AB antenatal OR AB antepartum OR AB ante-natal OR AB prenatal OR AB postnatal OR AB post-natal OR AB postpartum OR AB post-partum OR AB perinatal OR AB puerperium) AND (AB Randomized controlled trial? OR AB controlled clinical trial? OR AB clinical trial?) AND (AB Depression OR AB depressive OR AB mental OR AB psychological OR AB psychology)

*CINAHL*: (AB Physical exercise OR AB physical activity OR AB sport OR AB training OR AB endurance OR AB aerobic OR AB anaerobic OR AB resistance OR AB yoga) AND (AB Pregnant OR AB pregnancy OR AB antenatal OR AB antepartum OR AB ante-natal OR AB prenatal OR AB postnatal OR AB post-natal OR AB postpartum OR AB post-partum OR AB perinatal OR AB puerperium) AND (AB Randomized controlled trial? OR AB controlled clinical trial? OR AB clinical trial?) AND (AB Depression OR AB depressive OR AB mental OR AB psychological OR AB psychology)

*EMBASE*: ((Physical exercise or physical activity or sport or training or endurance or aerobic or anaerobic or resistance or yoga) and (Pregnant or pregnancy or antenatal or ante-natal or antepartum or prenatal or postnatal or post-natal or postpartum or post-partum or perinatal or puerperium) and (Randomized controlled trial? or controlled clinical trial? or clinical trial?) and (depressive or depression or mental or psychological or psychology)).ab.

*Cochrane Library:* #1 (Physical exercise):ti,ab,kw OR (physical activity):ti,ab,kw OR (sport):ti,ab,kw OR (training):ti,ab,kw OR (endurance):ti,ab,kw (Word variations have been searched)

#2 (aerobic):ti,ab,kw OR (anaerobic):ti,ab,kw OR (resistance):ti,ab,kw OR (yoga):ti,ab,kw (Word variations have been searched)

#3 #1 OR #2

#4 (Pregnant):ti,ab,kw OR (pregnancy):ti,ab,kw OR (antenatal):ti,ab,kw OR (ante-natal):ti,ab,kw OR (antepartum):ti,ab,kw (Word variations have been searched)

#5 (prenatal):ti,ab,kw OR (postnatal):ti,ab,kw OR (post-natal):ti,ab,kw OR (postpartum):ti,ab,kw OR (post-partum):ti,ab,kw (Word variations have been searched)

#6 (perinatal):ti,ab,kw OR (puerperium):ti,ab,kw (Word variations have been searched)

#7 #4 OR #5 OR #6

#8 (Randomized controlled trial?):ti,ab,kw OR (controlled clinical trial?):ti,ab,kw OR (clinical trial?):ti,ab,kw (Word variations have been searched)

#9 (Depression):ti,ab,kw OR (depressive):ti,ab,kw OR (mental):ti,ab,kw OR (psychological):ti,ab,kw OR (psychology):ti,ab,kw (Word variations have been searched)

#10 #7 AND #8 AND #9

*Web of Science*: Physical exercise OR physical activity OR sport OR training OR endurance OR aerobic OR anaerobic OR resistance OR yoga (Abstract) AND Pregnant OR pregnancy OR antenatal OR ante-natal OR antepartum OR prenatal OR postnatal OR post-natal OR postpartum OR post-partum OR perinatal OR puerperium (Abstract) AND Randomized controlled trial? OR controlled clinical trial? OR clinical trial?( Abstract) AND Depression OR depressive OR mental OR psychological OR psychology (Abstract)

PsycINFO: AB (Physical exercise OR physical activity OR sport OR training OR endurance OR aerobic OR anaerobic OR resistance OR yoga) AND AB (Pregnant OR pregnancy OR antenatal OR ante-natal OR antepartum OR prenatal OR postnatal OR post-natal OR postpartum OR post-partum OR perinatal OR puerperium) AND AB (Randomized controlled trial? OR controlled clinical trial? OR clinical trial?) AND AB (Depression OR depressive OR mental OR psychological OR psychology)

**Summary of Findings and Quality of Evidence**

| **Certainty assessment** | | | | | | | **№ of patients** | | **Effect** | | **Certainty** | **Importance** |
| --- | --- | --- | --- | --- | --- | --- | --- | --- | --- | --- | --- | --- |
| **№ of studies** | **Study design** | **Risk of bias** | **Inconsistency** | **Indirectness** | **Imprecision** | **Other considerations** | **[干预]** | **[对照]** | **Relative (95% CI)** | **Absolute (95% CI)** |  |  |
| **Type-comprehensive exercise** | | | | | | | | | | | | |
| 11 | randomised trials | serious | not serious | not serious | serious | none | 430 | 437 | - | SMD **0.58 SD lower** (0.9 lower to 0.25 lower) | ⨁⨁◯◯ Low | IMPORTANT |
| **Type-yoga** | | | | | | | | | | | | |
| 3 | randomised trials | not serious | not serious | not serious | serious | none | 63 | 60 | - | SMD **0.36 SD lower** (0.72 lower to 0 ) | ⨁⨁⨁◯ Moderate | IMPORTANT |
| **Type-walking** | | | | | | | | | | | | |
| 2 | randomised trials | not serious | not serious | not serious | serious | none | 12 | 13 | - | SMD **1.06 SD lower** (1.92 lower to 0.19 lower) | ⨁⨁⨁◯ Moderate | IMPORTANT |
| **Form-individual-based** | | | | | | | | | | | | |
| 7 | randomised trials | serious | not serious | not serious | not serious | none | 146 | 141 | - | SMD **0.36 SD lower** (0.6 lower to 0.13 lower) | ⨁⨁⨁◯ Moderate | IMPORTANT |
| **Form-group-based** | | | | | | | | | | | | |
| 5 | randomised trials | serious | not serious | not serious | serious | none | 228 | 237 | - | SMD **0.47 SD lower** (0.77 lower to 0.16 lower) | ⨁⨁◯◯ Low | IMPORTANT |
| **Form-individual+group-based** | | | | | | | | | | | | |
| 4 | randomised trials | serious | not serious | not serious | serious | none | 128 | 132 | - | SMD **0.91 SD lower** (1.8 lower to 0.03 lower) | ⨁⨁◯◯ Low | IMPORTANT |
| **Intensity-40-90 mins per week** | | | | | | | | | | | | |
| 7 | randomised trials | serious | not serious | not serious | not serious | none | 243 | 251 | - | SMD **0.51 SD lower** (0.73 lower to 0.29 lower) | ⨁⨁⨁◯ Moderate | IMPORTANT |

| **Certainty assessment** | | | | | | | **№ of patients** | | **Effect** | | **Certainty** | **Importance** |
| --- | --- | --- | --- | --- | --- | --- | --- | --- | --- | --- | --- | --- |
| **№ of studies** | **Study design** | **Risk of bias** | **Inconsistency** | **Indirectness** | **Imprecision** | **Other considerations** | **[干预]** | **[对照]** | **Relative (95% CI)** | **Absolute (95% CI)** |  |  |
| **Intensity-≥150 mins per week** | | | | | | | | | | | | |
| 6 | randomised trials | serious | not serious | not serious | serious | none | 190 | 187 | - | SMD **0.84 SD lower** (1.53 lower to 0.15 lower) | ⨁⨁◯◯ Low | IMPORTANT |
| **Duration-3~4weeks** | | | | | | | | | | | | |
| 2 | randomised trials | serious | not serious | not serious | serious | none | 71 | 70 | - | SMD **1.34 SD lower** (3.31 lower to 0.63 higher) | ⨁⨁◯◯ Low | IMPORTANT |
| **Duration-≥12 weeks** | | | | | | | | | | | | |
| 8 | randomised trials | serious | not serious | not serious | not serious | none | 281 | 271 | - | SMD **0.53 SD lower** (0.75 lower to 0.31 lower) | ⨁⨁⨁◯ Moderate | IMPORTANT |
| **Duration-6-9weeks** | | | | | | | | | | | | |
| 6 | randomised trials | serious | not serious | not serious | not serious | none | 153 | 159 | - | SMD **0.31 SD lower** (0.53 lower to 0.08 lower) | ⨁⨁⨁◯ Moderate | IMPORTANT |

**CI:** confidence interval; **SMD:** standardised mean difference


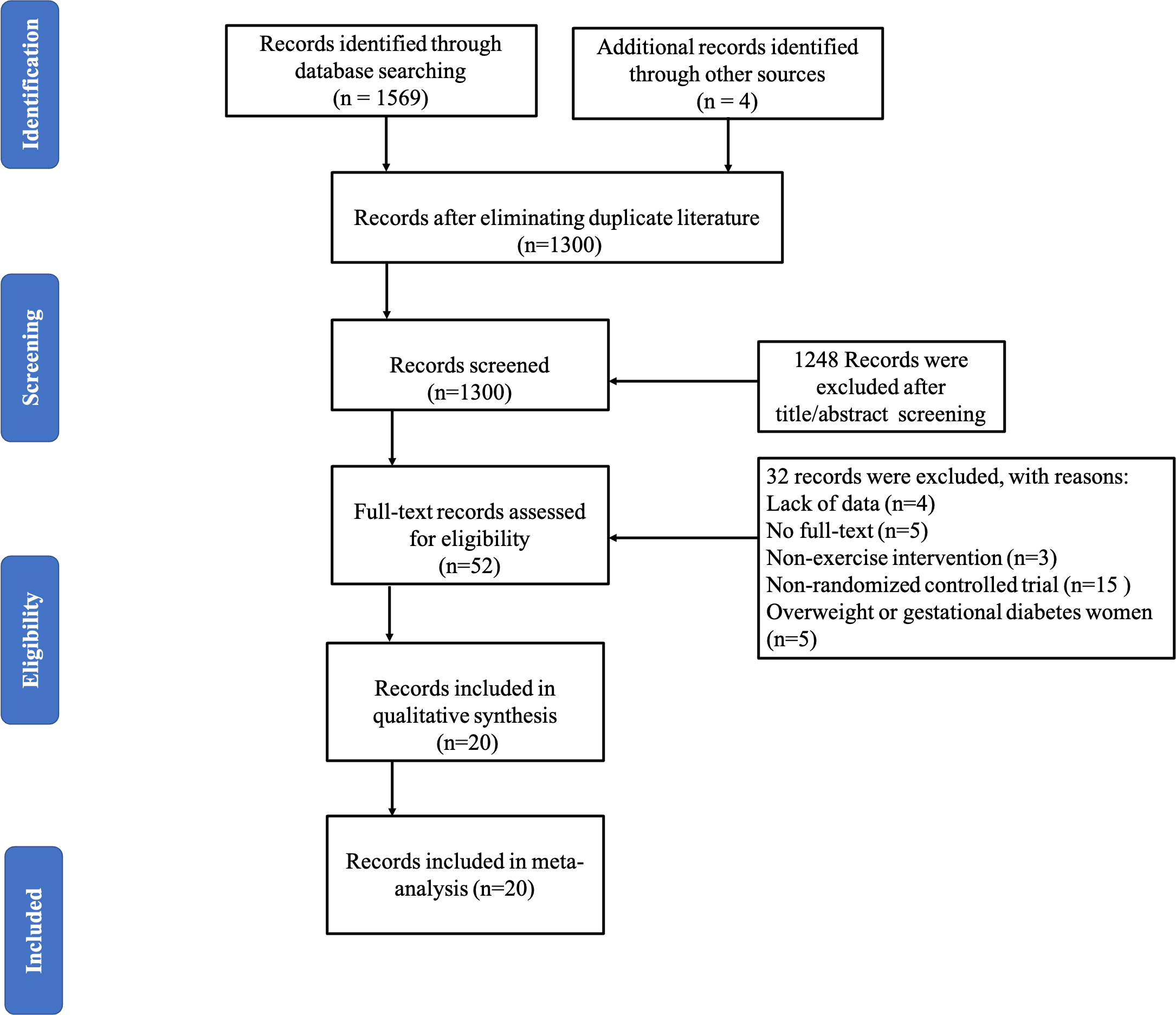


PRISMA flow diagram

PRISMA Checklist

| **Section and Topic** | **Item #** | **Checklist item** | **Location where item is reported** |
| --- | --- | --- | --- |
| **TITLE** | | |  |
| Title | 1 | Identify the report as a systematic review. | Page 1 |
| **ABSTRACT** | | |  |
| Abstract | 2 | See the PRISMA 2020 for Abstracts checklist. | Page 1 |
| **INTRODUCTION** | | |  |
| Rationale | 3 | Describe the rationale for the review in the context of existing knowledge. | Page 3-6 |
| Objectives | 4 | Provide an explicit statement of the objective(s) or question(s) the review addresses. | Page 6 |
| **METHODS** | | |  |
| Eligibility criteria | 5 | Specify the inclusion and exclusion criteria for the review and how studies were grouped for the syntheses. | Page 6-7 |
| Information sources | 6 | Specify all databases, registers, websites, organisations, reference lists and other sources searched or consulted to identify studies. Specify the date when each source was last searched or consulted. | Page 7 |
| Search strategy | 7 | Present the full search strategies for all databases, registers and websites, including any filters and limits used. | Page 8 |
| Selection process | 8 | Specify the methods used to decide whether a study met the inclusion criteria of the review, including how many reviewers screened each record and each report retrieved, whether they worked independently, and if applicable, details of automation tools used in the process. | Page 8 |
| Data collection process | 9 | Specify the methods used to collect data from reports, including how many reviewers collected data from each report, whether they worked independently, any processes for obtaining or confirming data from study investigators, and if applicable, details of automation tools used in the process. | Page 8 |
| Data items | 10a | List and define all outcomes for which data were sought. Specify whether all results that were compatible with each outcome domain in each study were sought (e.g. for all measures, time points, analyses), and if not, the methods used to decide which results to collect. | Page 8-9 |
|  | 10b | List and define all other variables for which data were sought (e.g. participant and intervention characteristics, funding sources). Describe any assumptions made about any missing or unclear information. | Page 8-9 |
| Study risk of bias assessment | 11 | Specify the methods used to assess risk of bias in the included studies, including details of the tool(s) used, how many reviewers assessed each study and whether they worked independently, and if applicable, details of automation tools used in the process. | Page 9 |
| Effect measures | 12 | Specify for each outcome the effect measure(s) (e.g. risk ratio, mean difference) used in the synthesis or presentation of results. | Page 9 |
| Synthesis methods | 13a | Describe the processes used to decide which studies were eligible for each synthesis (e.g. tabulating the study intervention characteristics and comparing against the planned groups for each synthesis (item #5)). | Page 9-10 |
|  | 13b | Describe any methods required to prepare the data for presentation or synthesis, such as handling of missing summary statistics, or data conversions. | Page 9-10 |
|  | 13c | Describe any methods used to tabulate or visually display results of individual studies and syntheses. | Page 9-10 |
|  | 13d | Describe any methods used to synthesize results and provide a rationale for the choice(s). If meta-analysis was performed, describe the model(s), method(s) to identify the presence and extent of statistical heterogeneity, and software package(s) used. | Page 9-10 |
|  | 13e | Describe any methods used to explore possible causes of heterogeneity among study results (e.g. subgroup analysis, meta-regression). | Page 9-10 |
|  | 13f | Describe any sensitivity analyses conducted to assess robustness of the synthesized results. | Page 9-10 |
| Reporting bias assessment | 14 | Describe any methods used to assess risk of bias due to missing results in a synthesis (arising from reporting biases). | Page 10 |
| Certainty assessment | 15 | Describe any methods used to assess certainty (or confidence) in the body of evidence for an outcome. | Page 9 |
| **RESULTS** | | |  |
| Study selection | 16a | Describe the results of the search and selection process, from the number of records identified in the search to the number of studies included in the review, ideally using a flow diagram. | Page 10 |
|  | 16b | Cite studies that might appear to meet the inclusion criteria, but which were excluded, and explain why they were excluded. | Page 10 |
| Study characteristics | 17 | Cite each included study and present its characteristics. | Page 11-13 |
| Risk of bias in studies | 18 | Present assessments of risk of bias for each included study. | Page 13 |
| Results of individual studies | 19 | For all outcomes, present, for each study: (a) summary statistics for each group (where appropriate) and (b) an effect estimate and its precision (e.g. confidence/credible interval), ideally using structured tables or plots. | Page11-12 |
| Results of syntheses | 20a | For each synthesis, briefly summarise the characteristics and risk of bias among contributing studies. | Page 14-16 |
|  | 20b | Present results of all statistical syntheses conducted. If meta-analysis was done, present for each the summary estimate and its precision (e.g. confidence/credible interval) and measures of statistical heterogeneity. If comparing groups, describe the direction of the effect. | Page 14-16 |
|  | 20c | Present results of all investigations of possible causes of heterogeneity among study results. | NA |
|  | 20d | Present results of all sensitivity analyses conducted to assess the robustness of the synthesized results. | Page 16 |
| Reporting biases | 21 | Present assessments of risk of bias due to missing results (arising from reporting biases) for each synthesis assessed. | Page 16 |
| Certainty of evidence | 22 | Present assessments of certainty (or confidence) in the body of evidence for each outcome assessed. | NA |
| **DISCUSSION** | | |  |
| Discussion | 23a | Provide a general interpretation of the results in the context of other evidence. | Page 16-22 |
|  | 23b | Discuss any limitations of the evidence included in the review. | Page 22-23 |
|  | 23c | Discuss any limitations of the review processes used. | Page 22-23 |
|  | 23d | Discuss implications of the results for practice, policy, and future research. | Page 24-25 |
| **OTHER INFORMATION** | | |  |
| Registration and protocol | 24a | Provide registration information for the review, including register name and registration number, or state that the review was not registered. | Page 6 |
|  | 24b | Indicate where the review protocol can be accessed, or state that a protocol was not prepared. | Page 6 |
|  | 24c | Describe and explain any amendments to information provided at registration or in the protocol. | NA |
| Support | 25 | Describe sources of financial or non-financial support for the review, and the role of the funders or sponsors in the review. | NA |
| Competing interests | 26 | Declare any competing interests of review authors. | Page 26 |
| Availability of data, code and other materials | 27 | Report which of the following are publicly available and where they can be found: template data collection forms; data extracted from included studies; data used for all analyses; analytic code; any other materials used in the review. | NA |
